# Molecular Evolution and Phylogenetic Analysis of Eight *COL* Superfamily Genes in Group I Related to Photoperiodic Regulation of Flowering Time in Wild and Domesticated Cotton (*Gossypium*) Species

**DOI:** 10.1371/journal.pone.0118669

**Published:** 2015-02-24

**Authors:** Rui Zhang, Jian Ding, Chunxiao Liu, Caiping Cai, Baoliang Zhou, Tianzhen Zhang, Wangzhen Guo

**Affiliations:** State Key Laboratory of Crop Genetics & Germplasm Enhancement, Hybrid Cotton R & D Engineering Research Center, MOE, Nanjing Agricultural University, Nanjing, China; USDA-ARS-SRRC, UNITED STATES

## Abstract

Flowering time is an important ecological trait that determines the transition from vegetative to reproductive growth. Flowering time in cotton is controlled by short-day photoperiods, with strict photoperiod sensitivity. As the *CO-FT* (*CONSTANS-FLOWER LOCUS T*) module regulates photoperiodic flowering in several plants, we selected eight *CONSTANS* genes (*COL*) in group I to detect their expression patterns in long-day and short-day conditions. Further, we individually cloned and sequenced their homologs from 25 different cotton accessions and one outgroup. Finally, we studied their structures, phylogenetic relationship, and molecular evolution in both coding region and three characteristic domains. All the eight *COLs* in group I show diurnal expression. In the orthologous and homeologous loci, each gene structure in different cotton species is highly conserved, while length variation has occurred due to insertions/deletions in intron and/or exon regions. Six genes, *COL2* to *COL5*, *COL7* and *COL8*, exhibit higher nucleotide diversity in the D-subgenome than in the A-subgenome. The *Ks* values of 98.37% in all allotetraploid cotton species examined were higher in the A-D and At-Dt comparison than in the A-At and D-Dt comparisons, and the Pearson’s correlation coefficient (r) of *Ks* between A vs. D and At vs. Dt also showed positive, high correlations, with a correlation coefficient of at least 0.797. The nucleotide polymorphism in wild species is significantly higher compared to *G*. *hirsutum* and *G*. *barbadense*, indicating a genetic bottleneck associated with the domesticated cotton species. Three characteristic domains in eight *COLs* exhibit different evolutionary rates, with the CCT domain highly conserved, while the B-box and Var domain much more variable in allotetraploid species. Taken together, *COL1*, *COL2* and *COL8* endured greater selective pressures during the domestication process. The study improves our understanding of the domestication-related genes/traits during cotton evolutionary process.

## Introduction

The taxonomic and evolutionary history of the cotton genus (*Gossypium*) extends back approximately 10 million years [1–3]. The cotton genus currently includes 50 species distributed in arid and semi-arid regions of the tropics and subtropics. Most of these species are diploid (n = 13), while five are allopolyploid (AD-genome; n = 26) [2,4]. *Gossypium tomentosum* is endemic to the Hawaiian Islands [5], while *G*. *mustelinum* is restricted to a relatively small region of northeast Brazil [6] and *G*. *darwinii* is native to the Galapagos Islands [7]. In addition to these three true wild species, *G*. *barbadense* and *G*. *hirsutum* are two cultivated allopolyploid species that have been independently domesticated over a vast geographical area, with a wealth of morphological forms spanning the wild to domesticated continuum [2,8–10]. Due to human-mediated influences and agronomic improvement, domesticated *G*. *barbadense* and *G*. *hirsutum* have been modified by parallel changes and exhibit extraordinary morphological variation, e.g., the loss of photoperiod sensitivity, transformation from perennial shrubs and small trees to more compact, highly productive annual plants, evolving seeds bearing vastly elongated, abundant, single-celled hairs, and a reduction in seed dormancy [11,12]. As a consequence of human selection and crop improvement, *G*. *hirsutum* has been domesticated for its dramatically high fiber yields and expanded planting area. Seven races of *G*. *hirsutum* have been identified to date, including ‘*yucatanense*’, ‘*punctatum*’, ‘*palmeri*’, ‘*latifolium*’, ‘*mariegalante*’, ‘*morrilli*’, and ‘*richmondii*’ [10]. Among these, ‘*latifolium*’ is considered to be the progenitor of the modern cultivated Upland cotton [13]. Semi-domesticated species of *G*. *barbadense* include several races such as ‘*peruvianum*’, ‘*vitifolium*’, and ‘*brasiliense*’; however, the origins of modern cultivated *G*. *barbadense* are complex and somewhat obscure [14]. In *Gossypium*, wild forms genetically close to the actual ancestors and domesticated species both exist, providing opportunities to study target genes selected during domestication by comparing both types of cotton and their parallel evolution [12].

Many crop species are subjected to similar evolutionary constraints and human involvement, which cause phenotypic changes that are common among crop species, namely, “domestication syndrome” (e.g., changes in flowering time) [12,15]. Flowering time is a common, important ecological trait that determines the transition from vegetative to reproductive growth and is regulated by four pathways including the autonomous, gibberellin, photoperiod, and vernalization pathways [16–19]. In particular, photoperiodic sensitivity is considered to be the most important factor in determining flowering time and thus ensures crop adaptation to specific growing seasons, cultivation areas, and natural environmental variation [20–22].

The *CONSTANS* (*CO*) transcription factor is a central regulator of the photoperiod pathway, which functions by mediating between the circadian clock and floral integrators [23,24], and *CONSTANS LIKE* (*COL*) genes are members of a recently identified family of plant zinc finger proteins. *CO* was first identified from an *Arabidopsis thaliana* mutant exhibiting late flowering, specifically under long-day photoperiodic conditions (LD) [25]. Subsequently, the *Hd1* (*Heading date 1*) gene of rice (*Oryza sativa*), which is homologous to *CO*, was also shown to be responsible for flowering time in rice [26]. Previous reports have shown that *COL* genes in several dicots, such as *Brassica napus BnCOa1* [27,28] and *Pharbitis nil PnCO*, could complement the function of *Arabidopsis CO* when introduced into a *co* mutant of *Arabidopsis*. Moreover, three *CO/Hd1* homologs of hexaploid wheat in monocots identified through sequence similarity analysis could complement the function of *Hd1* when transformed into a rice line deficient in *Hd1* [29]. The common function of these genes in different crops demonstrates that *CO* is involved in a conserved pathway regulating flowering in plants.

Since the release of a large number of publicly available sequences and the complete whole-genome sequences of some plants, genome-wide analyses of the *COL* gene family have been performed. There are 17, 17, and nine *COL* family members in *Arabidopsis*, rice, and barley, respectively [30–31]. Two conserved domains, including a zinc finger domain at the N-terminus that resembles B-boxes and a CCT (*CO*, *COL*, *TOC1*) domain at the C-terminus, are strictly conserved in these genes, while there is a more variable domain in the middle region. Phylogenetic analysis revealed that the *COLs* can be divided into four major groups. In detail, type I *COL* genes include two B-boxes and CCT domains, with a single intron located between the B-box and the CCT domains, which includes *AtCO* and *AtCOL1* to *AtCOL5* in *Arabidopsis* and *OsA* to *OsG* in rice. Type II *COL* genes, with only one B-box and a CCT domain with one intron, include *AtCOL*6 to *AtCOL8* and *AtCOL16* in *Arabidopsis* and *OsJ* to *OsL* in rice. Type III *COL* genes, with one full B-box, a second diverged zinc finger, and a CCT domain, include *OsM* to *OsP*, *Loc_Os06g01340*, *Loc_Os09g33550*, and *Loc_Os07g047140*, which are similar to *AtCOL9* to *AtCOL15* genes in *Arabidopsis*, containing three introns. *OsH* and *OsI*, which contain one intron, belong to type IV and are novel, as they lack B-box domains but have a *COL* CCT domain, this group of genes was recently designated the *CCT MOTIF FAMILY* (*CMF*) [22,30,32]. Mutants in B-boxes and CCT conserved domains display a severe late flowering phenotype [33]. Lagercrantz and Axelsson reported that the *COLs* evolve rapidly, particularly the variable domain in the middle region, which is the most diverged and most rapidly evolving, but there also are fixed residues that show significant conservation [34].

Flowering in an optimal condition could avoid stress damages and balance resource distribution, and further improve crop yield and quality [35]. The wild species of cotton is controlled by short-day photoperiods, with strict photoperiod sensitivity, while domesticated *G*. *barbadense* and *G*. *hirsutum* lose the photoperiod sensitivity [11,12]. So photoperiod sensitivity is considered as an important factor in determining flowering time in short-day photoperiods in cotton. Due to the conserved function of *CO* related to photoperiodic flowering in several plants and the limited information in cotton, we selected *CO* family members to study their structural and evolutionary characterization in *Gossypium*. Totally, we identified 23 putative *COL* genes in *G*. *raimondii* genome based on sequence data from *G*. *raimondii* (http://www.phytozome.net) [36] and divided these genes into three subfamilies, as reported by Griffiths et al. (2003) [30]. We focused on the eight genes in group I, which are also clustered in the same group with the *Arabidopsis CO* and rice *Hd1* flowering time loci. Diurnal expression were performed to analyze their function in respose to light and dark treatment, and we futher studied their sequence, structure, and molecular evolutionary rate variation in 25 cotton accessions, including the Old World diploids *G*. *herbaceum* L. and *G*. *raimondii* (which are considered to be extant relatives of the A- and D-genome diploid ancestors, respectively, of the allotetraploid lineage), three wild allotetraploid species, and 20 New World allotetraploid accessions (including 11 in *G*. *hirsutum*, with seven semi-domesticated species [races] and four cultivated accessions, and nine in *G*. *barbadense*, with three semi-domesticated species [races] and six cultivated accessions), as well as *Thespesia populneoides* (Roxb.) Kostel as a phylogenetic outgroup. We tested the footprints of selection sigatures for the eight *COL* genes by investigating the nucleotide diversity and neutrality test in allotetraploid cotton species. The study improves our understanding that the domestication-related genes have enhanced cotton adaptation and diversification during the evolutionary process, and the domestication and selection of *COL* genes might also contribute to the improvement of yield and quality in cotton.

## Materials and Methods

### Plant materials

Eight cotton genes among 23 *GrCOLs* were isolated from 25 different cotton accessions and one outgroup, which are listed in Table 1. One diploid A-genome species and one diploid D-genome species were chosen, which represent the best living models of the A- and D-genome donor, respectively. A total of 23 allopolyploid accessions involved in five cotton species were examined, including three wild allopolyploid species, 11 accessions from *G*. *hirsutum* species (seven semi-domesticated races and four cultivated accessions), and nine accessions from *G*. *barbadense* species (three semi-domesticated races and six cultivated accessions). *Thespesia populneoides* (Roxb.) Kostel was chosen as the outgroup. Cultivated *G*. *hirsutum* and *G*. *barbadense* accessions were sampled from the Jiangpu Experimental Station at Nanjing Agricultural University, Nanjing, Jiangsu, China. Other wild, semi-domesticated cotton species and the outgroup was collected from the National Wild Cotton Plantation at Hainan Island, China. All necessary permits for collecting the wild, semi-domesticated cotton species and the outgroup were obtained from the National Wild Cotton Plantation at Hainan Island, Cotton Research Institute, Chinese Academy of Agricultural Sciences, China. Genomic DNA was isolated from young leaves using methods reported previously [37].

**Table 1:** Cotton accessions used in the study with their geographic distribution in *Gossypium*.

No.	Species	Genome	Geographic distribution
1	*G*. *herbaceum* var. *Africanum*	A_1_	Africa
2	*G*. *raimondii* Ulbr	D_5_	Peru
3	*G*. *hirsutum* acc. TM-1	AD_1_	America
4	*G*. *hirsutum* cv. ZMS12	AD_1_	China
5	*G*. *hirsutum* cv. SM3	AD_1_	China
6	*G*. *hirsutum* cv. JM	AD_1_	China
7	*G*. *hirsutum* race *punctatum*	AD_1_	Mexico
8	*G*. *hirsutum* race *morrilli*	AD_1_	Mexico
9	*G*. *hirsutum* race *yucatanense*	AD_1_	Mexico
10	*G*. *hirsutum* race *richmondii*	AD_1_	Mexico
11	*G*. *hirsutum* race *marie-galante*	AD_1_	Mexico
12	*G*. *hirsutum* race *latifolium*	AD_1_	Mexico
13	*G*. *hirsutum* race *palmeri*	AD_1_	Mexico
14	*G*. *barbadense* cv. Hai7124	AD_2_	China
15	*G*. *barbadense* cv. Pima-1	AD_2_	America-Egypt
16	*G*. *barbadense* cv. Junhai	AD_2_	China
17	*G*. *barbadense* cv. Giza36	AD_2_	Egypt
18	*G*. *barbadense* cv. 3–79	AD_2_	America-Egypt
19	*G*. *barbadense* acc. Mit-Afifi	AD_2_	Egypt
20	*G*. *barbadense* race *peruvianum 1*	AD_2_	Peru
21	*G*. *barbadense* race *peruvianum 2*	AD_2_	Peru
22	*G*. *barbadense* race *brasiliense*	AD_2_	Brazil
23	*G*. *tomentosum* Nutt. Ex Seem.	AD_3_	Hawaii
24	*G*. *mustelinum* Miers ex Watt	AD_4_	Brazil
25	*G*. *darwinii* G. Watt	AD_5_	Galapagos Islands

### Identification of new *CO* family genes in cotton

COL protein sequences in *Arabidopsis* and rice referred to Griffiths et al. 2003 [22] and the Plant Transcription Factor Database (http://plntfdb.bio.uni-potsdam.de/v3.0/), reported by Wu et al. 2013 [30]. To identify *COL* transcription factor genes in the cotton genome, *CO* genes in *Arabidopsis* were used as queries to screen the Pfam database. Then, the Pfam database providing the B-box (PF00643) or CCT (PF06203) domain seed file was compared with the diploid cotton (*Gossypium raimondii*)_221_protein transcript database. The protein-coding genes with both B-box (PF00643) and CCT (PF06203) domains were manually examined as putative new members of *CO* in cotton (designated *GrCOLs*). Multiple alignments were then further performed with Cluster 1.83 and examined manually to confirm the correction. Finally, BLASTP was performed to search the diploid cotton (*G*. *raimondii*) genome database with an e-value cutoff of 1×10^–15^, and the genomic sequences of the *GrCOLs* were obtained.

### PCR amplification, cloning, and sequencing

Gene-specific PCR primers for eight *COLs* in group I were designed according to the predicted sequences in the diploid cotton (*G*. *raimondii*) genome database by Primer 5.0 (S2 Table). All PCR amplification products were extracted using an Axyprep DNA Gel Extraction Kit, cloned into the pMD19-T Vector (TaKaRa) according to the manufacturer’s instructions, and sequenced. In cases of apparent PCR-mediated recombination detection in allopolyploid cotton [38], at least 10 clones per gene were randomly sequenced, with at least three clones per subgenome, and these recombinant clones were omitted to confirm the sequence correction for each duplicated copy and to obtain the homeologs of both the A- and D-subgenomes by comparing sequences from their diploids using the Neighbor-Joining method in Clustal 1.83. The genomic sequences were compared with the cDNA sequences to determine the sizes of exons and introns.

### RNA isolation and qRT-PCR analysis

For diurnal expression pattern analysis, *G*. *hirsutum* acc. TM-1 were grown in LD (16h light/8h dark) or SD (8h light /16h dark) conditions respectively, and harvested the young leaves fully open every 4 h for 48h during the seeding period when the third leaf fully open, and put them in the liquid nitrogen immediately for use. Total RNA was extracted from leaves according to the method of Jiang and Zhang [39], and 10μl cDNA was synthesized using 500ng RNA with the HiScript Q RT SuperMix for qPCR (+gDNA wiper) (Vazyme), and the gene-specific primers used for qRT-PCR were designed by Primer 5.0 and are listed in (S2 Table). qRT-PCR (20μl reaction volume with 1μl cDNA, 0.5μM each gene-specific primer and FastStart Universal SYBR Green Master(ROX) (Roche) 10ul) were performed by ABI 7500 real-time PCR system. The *Histone3* (AF024716) gene (forward primer 5′-GAAGCCTCATCGATACCGTC-3′ and reverse primer squences 5′-CTACCACTACCATCATGG-3′ respectively) was used as the control. The expression level of *COL* genes was analyzed according to the relative quantification method [40].

### Data analysis

The corresponding subgenome sequences of each gene with the same order were combined, and the combined sequence data were used to conduct phylogenetic analyses using the Maximum Likelihood (ML) method provided by MEGA5.1, with 1,000 bootstrap replicates. A ML tree of CONSTANS-like proteins from *Arabidopsis*, rice, and cotton was also constructed to determine the evolutionary relationships between *GrCOL* gene family members and those of *Arabidopsis* and rice.

DnaSP 5.0 was used to estimate the total nucleotide diversity for the genomic sequence of each data set (π), and the nucleotide diversity of all site (π_total_), synonymous (π_s_) and nonsynonymous site(π_a_) for the entire coding region and separately for the B-box, Var, and CCT domains of each gene were also analyzed. The synonymous substitution rates (*Ks*) and nonsynonymous substitution rates (*Ka*) among 25 cotton accessions and one outgroup for the entire coding region of each gene, and neutral tests of Tajima’s D, Fu and Li’s D and F were also estimated by DnaSP 5.0 [41].

## Results

### Isolation and characterization of *COL* family genes in cotton

To identify genes encoding COL in the cotton genome, the primary HMM profiles using the B-box (PF00643) and CCT (PF06203) domains as the seed file, which were retrieved from the Pfam database [42], were used to search the diploid cotton (*Gossypium raimondii*)_221_protein transcript database (http://www.phytozome.net), and those with both domains (i.e., the candidate *COL* genes in *G*. *raimondii*) were manually selected.

**Fig 1:**
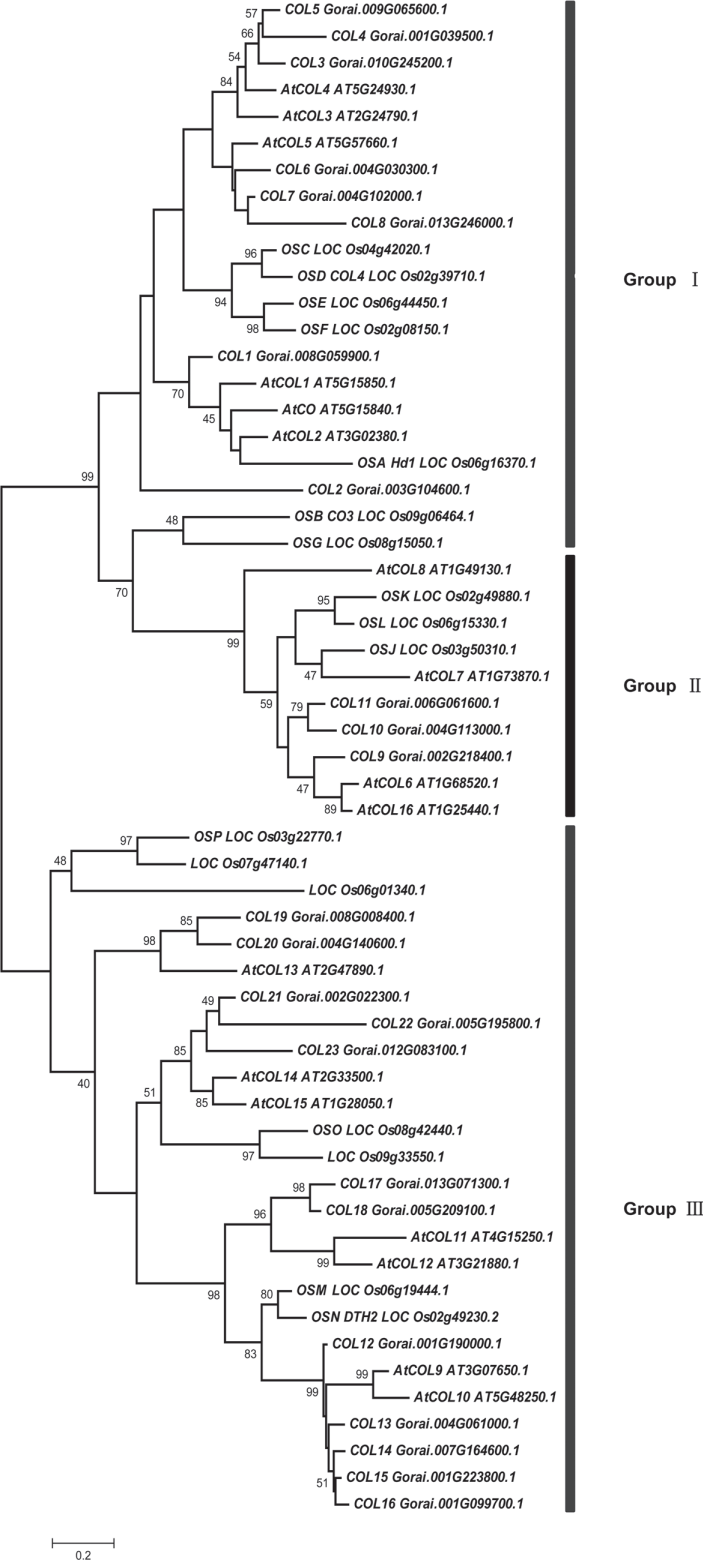
Maximum likelihood tree of *CONSTANS*-like proteins in *Arabidopsis*, rice and cotton. *CO*-like proteins in *Arabidopsis* and rice are based on Griffiths et al. (2003) and the Plant Transcription Factor Database (http://plntfdb.bio.uni-potsdam.de/v3.0/), and their amino acid sequences were obtained from the Plant Transcription Factor Database.

We identified 23 genes encoding both B-box and CCT domains, which were designated *COL1-23* (S1 Table). To clarify the phylogenetic relationship between *COL* family genes, we constructed phylogenetic trees using amino acid sequences of the COLs in *Arabidopsis*, rice, and cotton using the maximum likelihood (ML) method based on multiple alignment analyses. As shown in Fig. 1, three major clades are indicated in the tree, and COLs in cotton were classified into groups I, II, and III, which are similar to the groups identified for the dicot plant *Arabidopsis* and the monocot plant rice. These results indicate that divergence of COLs from different species occurred earlier than the divergence of monocots and dicots. There are eight genes in group I, which were predicted to encode two B-box and one CCT domain, except for *COL8*, encoding a protein with one intact B-box, one incomplete B-box, and one CCT domain. Three genes in group II were predicted to encode proteins containing one B-box and one CCT domain. The remaining 12 genes, which are in group III, encode one B-box, a second diverged zinc finger, and one CCT domain. Furthermore, we cloned the eight genes in group I, designated *COL1* to *COL8*, and studied these genes via phylogenetic and evolutionary analysis.

Using gene-specific primers (S2 Table), we performed full-length PCR cloning and sequencing of the eight genes in 25 cotton accessions and one outgroup (Table 1), which led to the identification of eight *COL* genes present in one copy in the diploid cotton species and two copies in the allotetraploids. The results of structural characterization of the eight genes in the 25 cotton accessions are summarized in Table 2. The eight genes are highly conserved, and their full-length genomic DNA sequences are ranging from 1,030 bp (*COL6*) to 1,611 bp (*COL1*), with exception of frame-shift mutation for 1bp deletion in few species. Multiple alignments of the genomic and cDNA sequences showed that all genes share the same one-intron structure. This intron ranges from 77 to 680 bp in length, with the longest intron present in *COL1*, *COL2*, and *COL8* compared with that of the other family members. For the same subgenome in different cotton accessions, insertion/deletion events occurred in introns or exon II of *COL2*, *COL6*, and *COL8*, leading to their length variation, while the remaining five genes had the same length in the same subgenomes of different cotton accessions. The structures of A- and D- homeologs from the same gene were further analyzed. Length differences were present in homeologs of *COL4* and *COL7*, which were caused by insertions/deletions in exon I or II. There are two distinct homeologs for all genes in each allotetraploid cotton accession, while there is a single type of *COL3* in the outgroup *Thespesia populneoides* (Roxb.) Kostel. Sequence information for these eight genes in the 25 cotton accessions and one outgroup has been submitted to GenBank (accession numbers: KM201660-KM202059).

**Table 2:** Structural analysis of eight COL genes in 25 cotton accessions.

Gene	Full length/length of ORF(bp)/number of AA/length of extron1/length of intron/length of extron2	Accession numbers
*COL1*	1611/1125/374/774/486/351	KM201660-KM201709
*COL2*	1^a^,14–22^a^, and 24–25^a^ At^b^:1547/1125/374/790/422/335;3–13^a^ At^b^:1550/1128/375/790/422/338;23^a^ At^b^:1502/1080/359/790/422/290;2 Dt^c^:1543/1122/373/790/421/332;3–25^a^ Dt^c^:1546/1125/374/790/421/335	KM201710-KM201759
*COL3*	1094/1017/338/699/77/318	KM201760-KM201809
*COL4*	23^a^ At^b^: 342/342/113/342;1^a^,3–22^a^,and 24–25^a^ At^b^:1071/981/326/697/90/284; Dt^c^:1090/999/332/697/91/302	KM201810-KM201859
*COL5*	1103/1008/335/693/95/315	KM201860-KM201909
*COL6*	At^b^:1033/945/314/626/88/319;2 Dt^c^:1030/942/313/626/88/316;3–23^a^ and 25^a^ Dt^c^:1031/942/313/626/89/316; 24^a^ Dt^c^:1032/942/313/626/90/316	KM201910-KM201959
*COL7*	At^b^:1206/1110/369/713/96/397; Dt^c^:1203/1107/368/710/96/397	KM202010-KM202059
*COL8*	14–19^a^ and 24^a^ At^b^: 1559/882/293/516/677/366;1^a^,3^a^,11^a^,13^a^ and 20–23^a^ At^b^:1565/888/295/516/677/372;12^a^ At^b^: 1568/888/295/516/680/372;14–22^a^ and 24^a^ Dt^c^:1519/882/293/516/637/366;9–10^a^,12–13^a^ Dt^c^:1548/888/295/516/660/372; 23^a^ Dt^c^:1549/891/296/516/658/375; 3–6^a^ Dt^c^:1552/894/297/516/658/378;8^a^ Dt^c^:1553/894/297/516/659/378;7^a^,11^a^ and 25^a^ Dt^c^:1554/894/297/516/660/378;2^a^ Dt^c^:1360/684/227/516/676/168	KM201960-KM202009

^a^ Code designations are the same as in Table 1.

^b^ At = A-subgenome from tetraploid cotton species.

^c^ Dt = D-subgenome from tetraploid cotton species.

### Diurnal expression pattern in light/dark cycles of the eight *COL* genes

**Fig 2:**
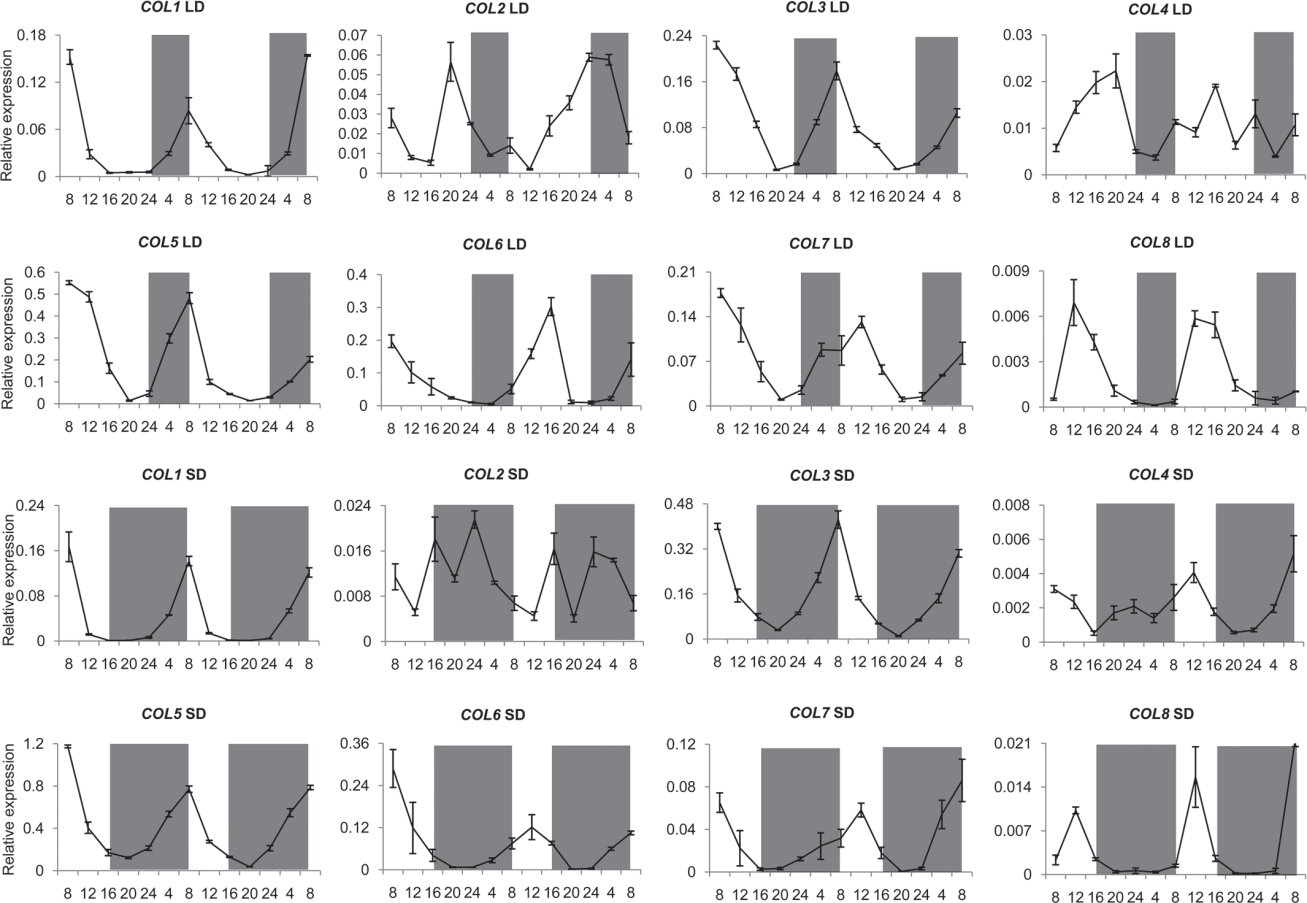
Diurnal expression patterns of eight *COL* genes in TM-1 leaves under LD or SD conditions. The X-axin represent time point (hours) and Y-axin indicates relative expression levels with cotton *histone3* (AF024716) as the control. Mean values ± SD were obtained from three biological repeats. The gray bars over each chart represent dark periods.

To examine the circadian rhythm of the candidate *COL* genes in cotton, we designed the gene-specific primers for qRT-PCR according to D-genome sequences (S2 Table), and investigated the expression level in the seedling leaves when the third leaf fully open under long-day (LD) (16h light/8h dark) or short-day (SD) (8h light/ 16h dark) condition respectively. The eight *COL* genes all showed diurnal expression patterns (Fig. 2). *COL1*, *COL3* and *COL5* exhibited the similar diurnal expression patterns under LD and SD conditions, the expression peaked in the dawn and started to decrease rapidly to the lowest at the end of light, then started to accumulate until the next dawn. *COL6* and *COL7* also had cycled with the light/dark induction treatment, but with the highest level 4 h later after the dawn and with lowest 8 h later. *COL8* expression started to accumulate after dawn with the peak 4 h later, and then declined quickly in the both photoperiodic conditions. *COL2* and *COL4* showed different expression patterns in the two photoperiodic conditions. The expression pattern of *COL4* was similar to *COL6* and *COL7* in SD condition, while there was no obvious diurnal expression pattern in LD condition. The expression of *COL2* started to accumulate at 4 h after dawn with expression peaking at dusk, and then declined during the dark in LD condition. However, *COL2* peaked twice in SD condition, its first peak occurred at the dusk, and reached the second peak 8h later. The diurnal expression patterns of the eight *COLs* suggest their conserved function in regulating the light signaling pathway in cotton.

### Eight *COL* homeologs from allotetraploid species showed independent evolution after polyploid formation

**Fig 3:**
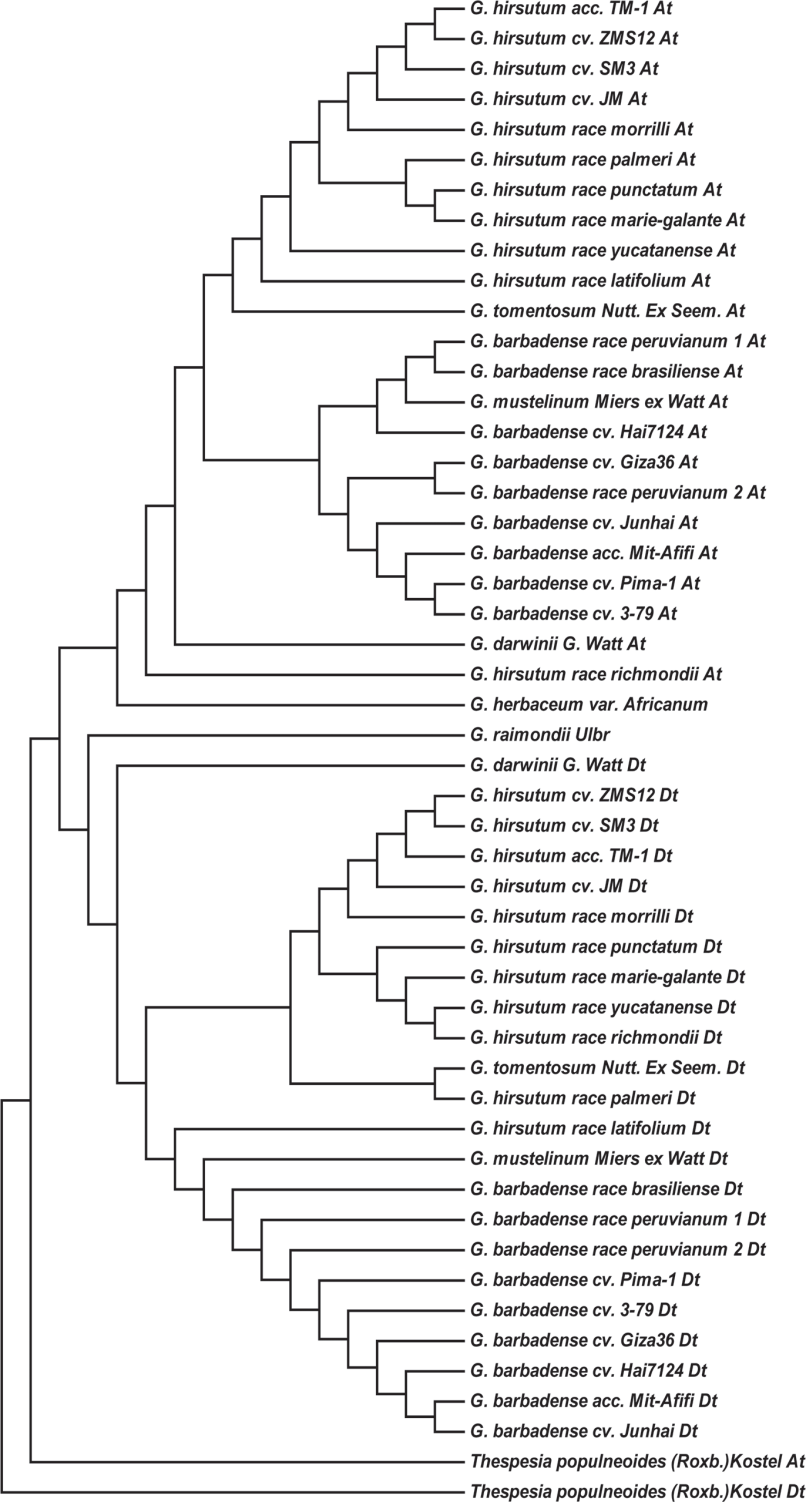
Phylogenetic tree based on the combined sequence of eight *CO*-like genes by the maximum likelihood method. Bootstrap values (%) based on 1000 replicates are indicated beside the nodes.

The sequences of the eight genes from the same subgenome were combined in order for 25 cotton accessions and one outgroup, and a phylogenetic tree was constructed using the ML method (Fig. 3). The outgroup *Thespesia populneoides* (Roxb.) Kostel was the most divergent member of this group and clustered into an individual clade, while the other members were divided into two principal clades; the A-genome and A-subgenomes in the tetraploid cotton accessions comprised one monophyletic clade, while the D-genome and D-subgenomes represented another monophyletic clade. Furthermore, the A-genome group was divided into two main subgroups; one included the A-subgenomes of *G*. *hirsutum* semi-domesticated, cultivated accessions and *G*. *tomentosum* and the other included the A-subgenomes of *G*. *barbadense* semi-domesticated, cultivated accessions and *G*. *mustelinum* species. *G*. *darwinii* and *G*. *hirsutum* race *richmondii* were clustered below in the A-genome group. Similarly, two main subgroups for *G*. *barbadense* and *G*. *hirsutum* were divided in the D-genome group, with the closest relationship between *G*. *hirsutum* and *G*. *tomentosum* and between *G*. *barbadense* and *G*. *mustelinum*. The exception was *G*. *darwinii*, the lone member of the D-genome group.

Using *G*. *herbaceum* and *G*. *raimondii* as controls for comparisons of their orthologs, we calculated the synonymous substitution rates (*Ks*) of each tested gene between orthologs (A vs. D, A vs. At, and D vs. Dt) and between homeologs (At vs. Dt) in the 25 accessions based on their coding regions (S3 Table). Of the 184 pairs compared (eight pairwise comparisons × 23 allotetraploid accessions) in allotetraploid species, the *Ks* values of 98.37% of the genes were higher in the A-D and At-Dt comparison than in the A-At and D-Dt comparisons. Furthermore, the Pearson’s correlation coefficient (r) of *Ks* between A vs. D and At vs. Dt also showed positive, high correlations, with correlation coefficients of at least 0.797 (Table 3).

**Table 3:** Correlation analysis between A-D and At-Dt comparisons for each allotetraploid accession.

Pairwise comparison	r of Ks
A-D vs 3 At-Dt	0.919**
A-D vs 4 At-Dt	0.919**
A-D vs 5 At-Dt	0.919**
A-D vs 6 At-Dt	0.894**
A-D vs 7 At-Dt	0.868**
A-D vs 8 At-Dt	0.922**
A-D vs 9 At-Dt	0.917**
A-D vs 10 At-Dt	0.842**
A-D vs 11 At-Dt	0.902**
A-D vs 12 At-Dt	0.939**
A-D vs 13 At-Dt	0.869**
A-D vs 14 At-Dt	0.890**
A-D vs 15 At-Dt	0.900**
A-D vs 16 At-Dt	0.888**
A-D vs 17 At-Dt	0.888**
A-D vs 18 At-Dt	0.889**
A-D vs 19 At-Dt	0.889**
A-D vs 20 At-Dt	0.905**
A-D vs 21 At-Dt	0.896**
A-D vs 22 At-Dt	0.903**
A-D vs 23 At-Dt	0.797*
A-D vs 24 At-Dt	0.873**
A-D vs 25 At-Dt	0.871**

Code designations are the same as in Table 1.

r: Correlation coefficient.

** Correlation is significant at P<0.01 (2-tailed).

* Correlation is significant at P<0.05 (2-tailed).

Taken together, these results suggest that A-D divergence for the eight *COLs* occurred well before the formation of the polyploids, and duplicated genes of A- and D- subgenomes from allotetraploid species evolve independently after the formation of the polyploids.

### Nucleotide diversity of the eight *COL*s showed different homoelogous evolutionary rate in allotetraploid species

Pairwise comparisons of nucleotide diversity (π) for the combined sequence of the eight *COL* genes and each gene between subgenomes within each allotetraploid accession was performed, respectively (Table 4). The average π value of the combined sequence in the D vs Dt (0.01051) were significantly greater than the value in A vs At (0.00586) (P = 4.9E-21). Among the 184 pairwise comparisons, 76.63% (141) harbored greater nucleotide diversity in the D-subgenome than that in the A-subgenome in the allotetraploid accessions. In detail, six genes, including *COL2* to *COL5*, *COL7* and *COL8*, showed significantly higher nucleotide diversity in the D-subgenome than in the A-subgenome of the allotetraploid accessions examined. However, *COL6* showed significantly higher nucleotide diversity in the A-subgenome than in the D-subgenome. There was no significant difference in the A vs At and D vs Dt in *COL1*. These results indicate that the eight *COLs* in group I harbor different evolutionary rates between homeologs of the allotetraploid accessions, and most genes of the D-subgenomes have been evolving more rapidly than those of the A-subgenomes.

**Table 4:** Estimates of nucleotide diversity of A vs At and D vs Dt for eight *COL* genes in cotton according to their genomic sequences.

Gene	A_1_-At	D_5_-Dt
combined sequence	0.00586	0.01051**
*COL1*	0.00605	0.00532
*COL2*	0.00624	0.00848**
*COL3*	0.0039	0.00827**
*COL4*	0.00678	0.01201**
*COL5*	0.00161	0.00584**
*COL6*	0.01124**	0.00536
*COL7*	0.0048	0.00575**
*COL8*	0.00631	0.02956**
average	0.00587	0.01007

Taxa include 23 allotetraploids accessions and the two genome donors to the allotetraploid accessions. At = A genome from the allotetraploid cotton species; Dt = D genome from the allotetraploid cotton species; D_5_ = *G*. *raimondii*; A_1_ = *G*. *herbaceum*.

** P<0.01

### Nucleotide diversity of the eight *COL*s showed different evolutionary rate in different cotton species and different domains

To further explore the domestication forces acting on allotetraploid species, we divided the tested allotetraploid accessions into three types, including tetraploid wild species, semi-domesticated and domesticated species of *G*. *hirsutum*, semi-domesticated and domesticated species of *G*. *barbadense*. Their nucleotide diversity (π) was estimated respectively for synonymous, nonsynonymous and the total sites of each data set with the ORF of each gene (Table 5). Generally speaking, the nucleotide diversity at synonymous substitution sites (π_s_) was significantly higher than that at non-synonymous substitution sites (π_a_) (0.00451 vs 0.00179)(P = 0.0001), and the three wild allotetraploid species possessed significally higher nucleotide diversity of π_total_ than *G*. *hirsutum* (0.00369 vs 0.00139) (P = 0.001), and *G*. *barbadense* (0.00369 vs 0.00035) (P = 7.3E-7), suggesting a genetic bottleneck associated with the domesticated cotton species.

**Table 5:** Nucleotide polymorphism and neutrality tests of eight *COL* genes in cotton according to their ORF sequences.

Gene	Accession	π_total_	π_s_	π_a_	TD	FD	FF
*COL1*At	total	0.00226	0.00479	0.00147	−2.21**	−3.62**	−3.73**
	*G*. *hirsutum*	0.00304	0.00734	0.0017	−2.00*	−2.33**	−2.54**
	*G*. *barbadense*	0.00059	0.00083	0.00052	−1.51	−1.68	−1.82
	wild	0.00178	0.00748	0	nd	Nd	nd
*COL1*Dt	total	0.00385	0.00752	0.00272	−1.01	0.06	−0.31
	*G*. *hirsutum*	0.00252	0.00477	0.00183	−1.34	−1.47	−1.63
	*G*. *barbadense*	0.00198	0.00396	0.00136	0.03	−0.29	−0.23
	wild	0.0077	0.01998	0.0039	nd	Nd	nd
*COL2*At	total	0.00335	0.00698	0.00232	−0.47	−1.52	−1.41
	*G*. *hirsutum*	0.00045	0	0.00059	−0.78	−0.33	−0.49
	*G*. *barbadense*	0.00089	0	0.00115	1.23	1.06	1.22
	wild	0.00681	0.01451	0.00462	nd	Nd	nd
*COL2*Dt	total	0.00232	0.00481	0.00161	−0.13	−1.57	−1.33
	*G*. *hirsutum*	0.00081	0.00144	0.00063	−1.79*	−2.13*	−2.30*
	*G*. *barbadense*	0	0	0	nd	Nd	nd
	wild	0.00533	0.01055	0.00385	nd	Nd	nd
*COL3*At	total	0.00274	0.00214	0.00294	0.10	−0.58	−0.44
	*G*. *hirsutum*	0.00097	0	0.00127	1.34	1.00	1.21
	*G*. *barbadense*	0.00055	0	0.00072	1.40	0.84	1.07
	wild	0.00393	0.00277	0.00431	nd	Nd	nd
*COL3*Dt	total	0.00282	0.00559	0.00198	0.20	−0.58	−0.41
	*G*. *hirsutum*	0.00136	0.00366	0.00066	−0.73	−0.76	−0.85
	*G*. *barbadense*	0	0	0	nd	Nd	nd
	wild	0.00459	0.00278	0.00517	nd	Nd	nd
*COL4*At	total	0.00198	0.00114	0.00224	−1.40	−2.41	−2.46
	*G*. *hirsutum*	0.00089	0.00158	0.00068	−1.32	−1.21	−1.39
	*G*. *barbadense*	0.00113	0	0.00148	0.03	0.23	0.20
	wild	0.00544	0.00291	0.00623	nd	Nd	nd
*COL4*Dt	total	0.00017	0	0.00023	−1.51	−2.13	−2.26
	*G*. *hirsutum*	0.00018	0	0.00024	−1.13	−1.29	−1.40
	*G*. *barbadense*	0	0	0	nd	Nd	nd
	wild	0.00067	0	0.00087	nd	Nd	nd
*COL5*At	total	0.00172	0.00287	0.0013	0.20	−0.95	−0.71
	*G*. *hirsutum*	0.00069	0	0.0009	0.04	−0.33	−0.27
	*G*. *barbadense*	0	0	0	nd	Nd	nd
	wild	0.00265	0.00833	0.00087	nd	Nd	nd
*COL5*Dt	total	0.00225	0.00434	0.00161	−1.05	−1.55	−1.63
	*G*. *hirsutum*	0.00152	0.00076	0.00176	−0.40	−0.08	−0.18
	*G*. *barbadense*	0	0	0	nd	Nd	nd
	wild	0.00463	0.01674	0.00087	nd	Nd	nd
*COL6*At	total	0.00236	0.00669	0.00106	−0.87	−2.25	−2.15
	*G*. *hirsutum*	0.00166	0.00399	0.00096	−1.40	−1.23	−1.44
	*G*. *barbadense*	0	0	0	nd	Nd	nd
	wild	0.00353	0.01209	0.00092	nd	Nd	nd
*COL6*Dt	total	0.00081	0.00308	0.00012	−0.83	−1.80	−1.76
	*G*. *hirsutum*	0.00019	0	0.00025	−1.13	−1.29	−1.40
	*G*. *barbadense*	0	0	0	nd	Nd	nd
	wild	0.00142	0.00612	0	nd	Nd	nd
*COL7*At	total	0.00191	0.00618	0.00066	0.37	−0.66	−0.42
	*G*. *hirsutum*	0.00033	0	0.00043	−1.43	−1.66	−1.80
	*G*. *barbadense*	0.0005	0	0.00065	1.40	0.84	1.07
	wild	0.0018	0.00529	0.00078	nd	nd	nd
*COL7*Dt	total	0.00274	0.00564	0.00188	−1.08	−2.20	−2.18
	*G*. *hirsutum*	0.00312	0.0046	0.00267	−1.44	1.71	−1.86
	*G*. *barbadense*	0	0	0	nd	nd	nd
	wild	0.0012	0.00264	0.00078	nd	nd	nd
*COL8*At	total	0.00341	0.00671	0.00241	0.37	−1.08	−0.76
	*G*. *hirsutum*	0.00119	0.00203	0.00093	−1.46	−1.44	−1.63
	*G*. *barbadense*	0	0	0	nd	nd	nd
	wild	0.00378	0.00647	0.00299	nd	nd	nd
*COL8*Dt	total	0.00394	0.00366	0.00405	0.57	0.51	0.62
	*G*. *hirsutum*	0.00324	0.00637	0.00227	1.59	1.33^#^	1.57^#^
	*G*. *barbadense*	0	0	0	nd	nd	nd
	wild	0.00378	0	0.00498	nd	nd	nd
average	total	0.00241	0.00451	0.00179	−0.54725	−1.39592	−1.33363
	*G*. *hirsutum*	0.00139	0.00228	0.00111	−0.83652	−0.72036	−1.02529
	*G*. *barbadense*	0.00035	0.00030	0.00037	0.16132	0.06295	0.09409
	wild	0.00369	0.00742	0.00257	nd	nd	nd

TD, the Tajima’s D; FD, Fu and Li’s D; FF, Fu and Li’s F.

nd, not determined (not implemented yet).

# *P*<0.1

* *P*<0.05

** *P*<0.01.

As reported by Robson et al. (2001) [33] and Griffiths et al. (2003) [30], the B-box and CCT domains are two conserved domains of CO proteins that are required for the promotion of flowering. To further explore the evolutionary rate of the three characteristic domains, we further analyzed the nucleotide diversity of the three domains of each data set for the eight *COL* genes respectively (Table 6). The nucleotide diversity of most genes was significantly lower in the CCT domain, while the B-box domain and the Var domain possessed relatively high rates of replacement substitutions. There were no diffrences in π between B-box and the Var domain (P = 0.285), and the two domains evolved signifacally faster than the CCT domain (0.00284 vs 0.00119, P = 0.028 for B-box and 0.00243 vs 0.00119, P = 0.014 for Var domain, respectively). These results demonstrate that the B-box and Var domains have been quite variable, while the CCT domain is highly conserved in sequence and function among 25 cotton accessions.

**Table 6:** Nucleotide polymorphism of B-box, Var and CCT domains for eight *COL* genes in allotetraploid cotton.

Gene	B-box	Var	CCT
*COL1*At	0.00104	0.00213	0.00251
*COL1*Dt	0.0016	0.00493	0.00576
*COL2*At	0.00696	0.00314	0
*COL2*Dt	0.00311	0.00269	0
*COL3*At	0.00406	0.00145	0.00067
*COL3*Dt	0.00357	0.002	0.00135
*COL4*At	0.00435	0.0014	0.00067
*COL4*Dt	0.00035	0.00016	0
*COL5*At	0.00069	0.00285	0
*COL5*Dt	0.0027	0.00149	0.00202
*COL6*At	0.00073	0.00306	0.00533
*COL6*Dt	0.00073	0.00151	0
*COL7*At	0.00315	0.00236	0
*COL7*Dt	0.00248	0.00376	0
*COL8*At	0.0072	0.00022	0.00067
*COL8*Dt	0.00267	0.00579	0
average	0.00284	0.00243	0.00119

### Neutrality tests of the eight *COL* genes reveal three selected genes during domestication

To test the departure from neutrality, Tajima’s D (1989) and Fu and Li’s D and F (1993) [43–44] were estimated to test whether the nucleotide polymorphism data of the eight *COL* genes fit the neutral model (Table 5). We showed that both *COL1* A-subgenome and *COL2* D-subgenome in *G*. *hirsutum* significantly deviated from the neutral expectation with a negative value, indicating an excess of low frequency alleles. And the negative values are consistent with the possibility of recent positive selection in *G*. *hirsutum*. Fu and Li’ D and F were significantly positive in *COL8* D-subgenome of *G*. *hirsutum* at P<0.1, this result suggest that the allele of *COL8* maintained a high frequency variants and might experience balance selection [45–46]. Taken together, *COL1*, *COL2* and *COL8* endured greater selective pressures during the domestication process.

## Discussion

### The homeologs of eight *COL* genes are evolving independently at the allopolyploid level

Allotetraploids originate from an interspecific hybridization event between diploid A- and D-genome species. Here, we performed ML analysis of the eight genes among 26 accessions, including 25 cotton accessions and one outgroup, to help elucidate the relationship between the homeologs at the allopolyploid level. The phylogenetic analysis showed that the outgroup *Thespesia populneoides* (Roxb.) Kostel was quite distant from the other allotetraploid cotton species and clustered into an individual clade, while the others were divided into two major clades, each containing the At or Dt subgroup with their corresponding diploid ancestral species. The results show that homeologs of the eight genes are evolving independently in the tetraploid accessions examined, including wild, semi-domesticated, and cultivated species. Furthermore, 98.37% of the *Ks* values were higher in the A-D and At-Dt comparisons than in the A-At and D-Dt comparisons, and the Pearson’s correlation coefficient for the A-At and D-Dt comparisons of the eight genes of the diploid and all of the allotetraploid accessions exhibited a significant positive correlation (*r* ^*2*^ = 0.797). This observation indicates that the A-D divergence occurr well before the formation of the polyploids, and duplicated genes of At and Dt of eight *COL* genes from allotetraploid species evolve independently after the formation of the polyploids. These results are in agreement with the results of previous reports [47–49]. From the study, *G*. *tomentosum* (from the Hawaiian Islands) had a closer relationship with *G*. *hirsutum*, while *G*. *mustelinum* was closer to *G*. *barbadense* than to *G*. *darwinii*. Similarly, the D and Dt clade yielded similar results to those of the A and At clade. These results are also largely in agreement with those of previous studies [2,5].

### COL transcription factors have conserved functions among different plant species

COL transcription factors play important roles in regulate flowering time in the photoperiod signaling pathway, which coordinates light and circadian clock inputs (primarily in leaves) to induce the expression of the florigen gene *FLOWERING LOCUS T* (*FT*) [50–51]. These proteins are widely present among species, from lower plants such as mosses [52–53] to algae (which exhibit strong photoperiod responses [54–55]) to higher flowering plants including monocots and dicots. These transcription factor genes include *CO* in *Arabidopsis*, *Hd1* in rice, and its homologs in barley, ryegrass, sugar beet, and soybean [25,26,56–59]. The *CO-FT* module is conserved in all known plant species, although it has different modes of action in different species. CO promotes the expression of *FT* under LD conditions in *Arabidopsis thaliana* [25,33], while Hd1, the ortholog of CO in rice, functions in the promotion of *Hd3a* (the *FT* ortholog) expression under SD conditions and as a repressor under non-inductive long day conditions [50,60]. CO is a central regulator of the photoperiod pathway, triggering the production of the mobile florigen hormone FT, which induces flower differentiation. The homologs of *COL3* and *COL5* have previously been cloned in cotton, and qRT-PCR analysis shows that the expression of the *COL5* homolog is controlled by daily oscillations and exhibits a diurnal rhythm, with higher expression levels observed in the dark than in the light [61]; this expression pattern is similar to that of *CO* in *Arabidopsis* and *COL* in other plants [22,31,56,62,63]. *COL* genes in group I of cotton harbored two B-box and a CCT conserved domains with the same to that in other plants, and expression analysis indicated that all the eight genes showed a diurnal rhythm expression pattern in TM-1. *COL1*, *COL3*, and *COL5*-*COL7* showed similar diurnal expression patterns under both LD and SD conditions, and the expression peak were present in the dawn or 4h later, and declined rapidly to the lowest until dusk, with similar to *AtCOL1* and *AtCOL2* in *Arabidopsis*, *GmCOL1* and *GmCOL2* in soybean, *OsB*, *OsE* and *OsD* in rice [31,33,56]. *COL8* showed similar diurnal expression pattern with *OsG*, which was also a special gene with internal deletion of the second B-box domain [31]. *COL4* was one unique gene that the diurnal expression pattern were more evident under SD than in LD condition just like *ZCN8* in maize[64], and *COL4* might perceive SD signal in TM-1, but not responsive to LD regulation. The *COL2* expression in LD condition peaked once per 24h-period and twice in SD condition. The diurnal expression analysis indicated that the *COL* gene family in group I was potentially involved in regulating the light signaling pathway or photoperiodic flowering in cotton as other plants, but more detailed functional analyses are needed for further study.

### Selection signatures of eight *COL* genes in coding region and domains in the allotetraploid species

Nucleotide polymorphisms of the eight *COL* genes show that most *COL* genes in wild allopolyploid species possess significantly higher nucleotide diversity than that of *G*. *hirsutum* and *G*. *barbadense*, this reduction of diversity could result from genetic bottlenecks during various stages of domestication. The limited genetic diversity of cultivated *G*. *hirsutum* had been observed in previous studies [65–66]. The neutrality test showed that *COL1* A-subgenome and *COL2* D-subgenome of *G*. *hirsutum* significantly deviated from zero with a negative value, implying an excess of low frequency alleles. This was consistent with the possibility of recent positive selection in *G*. *hirsutum* [3,11]. *COL1* displays diurnal expression patterns with similar to *AtCOL1* and *AtCOL2* in *Arabidopsis*, the result indicated that *COL1* in cotton may play conserved function in light input pathway but not affect flowering time [62]. While *COL2*, orthologous to *Hd1* in rice, exhibits distinct diurnal expression in LD and SD conditions, indicating that *COL2* was potentially regulating photoperiodic flowering in cotton, with similar function as *Hd1* [26]. So, *COL1* and *COL2* genes were the potential target of positive selection in light signal or photoperiodic flowering pathway of *G*. *hirsutum*. Especially, nucleotide diversity of *COL2* D-subgenome of *G*. *hirsutum* is approximately sixfold lower than the wild allopolyploid species, so *COL2* is expected to be the better selected CO gene in cotton. Interestingly, *COL8*, with one intact B-box domain similar to that of *OsB/OsCO3* and *OsG* [30], evolved faster among the tested eight genes. *OsB/OsCO3* was reported to regulate negatively the photoperiodic flowering in rice [67], and *COL8* showed similar diurnal expression pattern with *OsG* [31]. So *COL8* might also involve in the photoperiodic flowering in cotton. The neutrality tests of *COL8* D-subgenome were significantly positive with P<0.1 in *G*. *hirsutum*, indicating an excess of higher frequency alleles, and balancing selection is expected to act on *COL8* [45]. Higher frequency variants in *COL8* D-subgenome may contribute to satisfy the need of multiple environments and better adaptation of cotton, and promote the evolution of photoperiod sensitivity in *G*. *hirsutum*. Taken together, selection acted on the three potential target *COL* genes in *G*. *hirsutum* might be responsibe for the wide adaptation of *G*. *hirsutum* [2]. In other plants such as rice and maize, selection of *COL* homologs appears to be common during parallel adaptation [12,22,68–70].

CO is a typical transcription factor with three characteristic domains, including the B-box, Var domain, and CCT domain, which indicates that it is a unique type of transcriptional regulator present only in the plant kingdom [25]. *COL* genes within the *Brassicaceae* family are evolving rapidly, and different domains in the *COL* genes are heterogeneous [34]. We analyzed the nucleotide diversity of B-box domain, the Var domain and the CCT domain in cotton respectively. The results suggested that the nucleotide diversity of most genes were significantly lower in the CCT domain, indicated that the CCT domain is highly conserved, possibly due to high functional evolutionary constraints acting on this domain. Natural variation within genes with CCT domains has previously been reported, including *COL*, *PRR* (*PSEUDO RESPONSE REGULATORs*), and *CMF* genes, which are critical to the control of plant flowering [24–26,32,71–73]. The CCT domain shows homology to the NF-YA1/2 domains of HAP2, which help form the trimeric CO/At HAP3/At HAP5 complex and bind to CCAAT boxes in eukaryotic promoters to regulate flowering of *Arabidopsis* through the expression of *FT* [74], as well as interacting with the ubiquitin ligase COP1 [75] and nuclear localization signal [30,33]. Therefore, the strong conservation of the CCT domain is thought to be necessary for its role in the control of photoperiodic flowering. B-box domain is involved in DNA binding and protein-protein interactions, as plants with mutations in this region display severe late flowering phenotypes [33,76]. Most genes with B-box domains display a divergent diurnal expression pattern, indicating that this domain functions in the light signaling pathway [31,61,62,77]. The Var domain, with a lower degree of conservation in amino acid sequence among the COLs, activates transcription, as demonstrated by yeast-two hybrid assays [78], although its fixed residues are significantly conserved. It is recently shown that *DTH2*, which encodes a COL protein in rice, and two functional nucleotide polymorphisms (FNPs) in the B-box and Var domain, respectively, are associated with the changes in flowering time and increased reproductive fitness that have occurred during the northward expansion of rice cultivation [22]. In this study, the B-box and the Var domain evolve signifacally faster than the CCT domain among the eight *COL* genes, indicating the two domains endure relax evolutionary constraint, and may be associated with the changes in flowering time of cotton. Higher nucleotide diversity in the two domains may enable cotton to form a diversity of habitats to adapt the variable environments and expansion of the cultivation area.

## Conclusions

*CO-FT* is conserved and plays important roles in the photoperiodic regulation of flowering time in plants. We revealed that eight *COL* homeologs from allotetraploid accessions have evolved independently after polyploid formation. *COL1*, *COL2* and *COL8* are potential selected genes during domestication, with strong conservation on the CCT domain and great diversity on the B-box and Var domains. This study provides valuable information that increases our understanding of the dynamic evolutionary of the *COL* gene family in cotton and the potential target *COL* genes during the domestication and adaptation of cotton.

## Supporting Information

S1 Table Genome-wide *COL* genes analysis in *Gossypium*. (XLS)

S2 Table The primer pairs used for amplifying the eight *COL* genes sequences and qRT-PCR analysis. (XLS)

S3 Table Synonymous and nonsynonymous substitution rates and their ratio for genes in different AD-genome accessions and outgroup (A vs. D, A vs. At, D vs. Dt, and At vs. Dt) (Ka/Ks/Ka:Ks ratio). (XLS)
